# MALAMA: Cultivating Food Sovereignty through Backyard Aquaponics with Native Hawaiian Families

**DOI:** 10.3390/genealogy8030101

**Published:** 2024-08-07

**Authors:** Jane J. Chung-Do, Phoebe W. Hwang, Ilima Ho-Lastimosa, Ikaika Rogerson, Kenneth Ho, Kau’i DeMello, Dwight Kauahikaua, Hyeong Jun Ahn

**Affiliations:** 1 Office of Public Health Studies, Thompson School of Social Work and Public Health, University of Hawai’i at Mānoa, Honolulu, HI 96822, USA; 2 Ke Kula Nui O Waimānalo, Waimānalo, HI 96795, USA; 3 Department of Psychiatry, John A. Burns School of Medicine, University of Hawai’i at Mānoa, Honolulu, HI 96817, USA; 4 Department of Tropical Plants and Social Sciences, College of Tropical Agriculture and Human Resources, University of Hawai’i at Mānoa, Honolulu, HI 96822, USA; 5 Waimānalo Community Partner, Waimānalo, HI 96795, USA; 6 Department of Quantitative Health Sciences, John A. Burns School of Medicine, Honolulu, University of Hawai’i at Mānoa, Honolulu, HI 96813, USA

**Keywords:** food sovereignty, food security, Native Hawaiian, Indigenous, community health, family, nutrition, sustainability, aquaponics, culture/ethnicity

## Abstract

Native Hawaiians were a healthy and robust population who developed a sophisticated food system that was dismantled by colonization. Currently, Native Hawaiians face pervasive health disparities due to the limited access to healthy foods and lifestyles. This study pilot tested a family-based community-driven intervention called MALAMA, which teaches families to build and use a backyard aquaponics system to grow their own food. A total of 21 participants from 10 families completed a three-month curriculum that included a series of hands-on workshops. Participant attendance was recorded and participants completed a behavioral health questionnaire as well as provided clinical indicators at three time points. They also attended a focus group at the end of the curriculum. There was a high level of engagement and no participant attrition. Fruit consumption among all participants significantly increased and there were favorable trends in blood pressure and fish and vegetable consumption. No significant differences were found in the other clinical indicators. Participants found MALAMA to be highly culturally acceptable and identified multiple benefits. Community-driven solutions, such as MALAMA, may be a promising approach to addressing pervasive health disparities and promoting health equity in minority and Indigenous communities.

## 1. Introduction

Native Hawaiian ancestors were healthy people with a robust food system. Shaped by island geography, Native Hawaiians created the ahupua’a system, which uses a wedge-shaped area of land running from the mountain to the sea, following the natural boundaries of the watershed (Ahupua’a 2022). Each ahupua’a contained the resources for food, including fish, salt, vegetables, fruit, and other plants. With western colonization came the privatization of land, mass plantations, and militarization that fragmented this traditional and sustainable food system, which has contributed to the pervasive health and socioeconomic disparities among Native Hawaiians seen today (Kame’eleihiwa 1992).

Today, the health of Native Hawaiians reflects the historical trauma they have endured to their identities and land (Brave Heart 2003; Kaholokula et al. 2009). The State of Hawai’i has one of the lowest obesity rates (22.1%) in the nation, but 38.7% of Native Hawaiian adults are obese. This is higher than the rate for any other ethnic group in Hawai’i (Hawaii-BRFSS 2022). Hawaiians also face high rates of type 2 diabetes (22.0%) and cardiovascular disease (18.8%) and are 130% more likely to die from diabetes and 68% more likely to die from heart disease compared to the state average (Look et al. 2020). These disparities are compounded by food insecurity issues. Hawai’i’s residents face the highest food costs in the U.S., as 90% of the food supply is imported (OP–DBEDT 2012). Furthermore, Native Hawaiians tend to live in neighborhoods with limited availability of healthy food options (Lee et al. 2012; Mau et al. 2008).

Despite these challenges, Native Hawaiian communities have demonstrated resilience and strengths through dedicated cultural practitioners and community organizing. Since the 1970s, the Native Hawaiian Renaissance Movement has made tremendous efforts and strides in revitalizing Native Hawaiian cultural practices, such as the Hawaiian language, oceanic voyaging, food cultivation, and land and ocean restoration (Goodyear-Kaopua et al. 2014). Many rural Native Hawaiian communities, such as the community of Waimānalo, have been instrumental in retaining and promoting cultural practices. Waimānalo is a rural community located on the eastern side of the island of O’ahu. It is home to over 6000 residents with one-third being Native Hawaiian, which is twice the state average (United States Census Bureau 2022). Although it is a medically underserved population with high levels of food insecurity and socioeconomic challenges (Hawaii Department of Health 2019), this close-knit community possesses numerous strengths. Waimānalo is home to many cultural practitioners and leaders, which has helped sustain Hawaiian cultural practices, perpetuate the community’s reputation for community advocacy and organizing, and foster a large number of Native Hawaiian grassroots organizations (Ho-Lastimosa et al. 2020).

God’s Country Waimānalo (GCW) is an example of a Native Hawaiian grassroots organization in Waimāmalo. GCW’s mission is to propagate and perpetuate Hawaiian culture by teaching community members to grow their own food through multiple methods of home food production, including backyard aquaponics. Aquaponics has been found to be attractive to the Hawaiian community because it mimics the ahupua’a system, where fish and plants symbiotically grow in a sustainable and ecological system (Beebe et al. 2020). Aquaponics systems optimize water and nutrient use efficiency by combining hydroponics (soilless horticulture) and aquaculture (raising fish in tanks), which uses a fraction of the water and nutrients compared to traditional terrestrial systems (Tokunaga et al. 2013). Since 2009, GCW has partnered with students and researchers from the University of Hawai’i who helped collect data from these efforts by conducting focus groups, interviews, and surveys with the participants.

Building on the longstanding relationships between the community leaders from GCW and the wider Waimānalo community with public health researchers from the University of Hawai’i, a grant proposal was collectively developed and submitted to conduct a pilot clinical health study on this community-driven backyard aquaponics initiative as a culturally-grounded solution to promote Native Hawaiian health. The backyard aquaponics project was named the MALAMA program. As is common in ‘ōlelo Hawai’i (Hawaiian language), the meaning of MALAMA is layered. MALAMA is an acronym for “Mini Ahupua’a for Lifestyle And Mea’ai (food) through Aquaponics.” The word, “mālama,” also means to “take care of, tend to, preserve, and protect”. MALAMA seeks to teach families how to take care of themselves, their families, their community, as well as the food they cultivate and consume. MALAMA aims to perpetuate the Hawaiian concept of land stewardship, mālama ‘āina, which aligns with the ‘ōlelo no’eau (Hawaiian proverb), *E Malama ‘oe I ka ‘Āina, e Malama ka ‘Āina ia ‘oe*, which means, “Take care of the land and the land will take care of you”.

Based on the previous work of GCW, seven hands-on family-based workshops were standardized and delivered over three months to a hui (cohort) of 10 families (Ho-Lastimosa et al. 2019). The workshops were generally 3 h long and focused on increasing participants’ knowledge and skills related to gardening, fish help, aquaponics technology, nutrition, traditional medicine, etc. Participants also came together for a weekend to build their aquaponics systems and helped each other install them in each of the participants’ homes. The workshops were designed so the participants can get to know one another and build relationships with one another as neighbors. Five peer mentors called Lima Kōkua, who have previous experience with aquaponics, were also enlisted to support the participants.

The goal of this pilot study was to test the feasibility and acceptability of the MALAMA program as a public health intervention to promote healthy eating and address the pervasive health disparities that Native Hawaiians currently face. To test the feasibility of the MALAMA program as a health intervention, a multi-methods design was utilized. To determine feasibility, participant attendance was tracked to assess recruitment and retention. A behavioral health questionnaire and clinical measures were collected to determine the feasibility of carrying out this type of data collection and if behavioral eating changes could be detected. Clinical changes were not expected due to the short duration of the intervention and the research design. Acceptability was determined through the qualitative focus group. MALAMA is the first known aquaponics health intervention to date.

## 2. Materials and Methods

### 2.1. Community Partnership

The MALAMA study is currently a program under Ke Kula Nui O Waimānalo (KKNOW), which stemmed from the work by GCW. KKNOW is a grassroots nonprofit organization that was founded in 2017 by community leaders and partners of Waimānalo (Ho-Lastimosa et al. 2020). The goal of KKNOW is to cultivate resilience throughout the community of Waimānalo and other Native Hawaiian communities through community-led programs that support economic, cultural, and environmental self-sustainability. These community-led programs aim to leverage ancestral wisdom to promote multigenerational learning and build relationships within Hawaiian families and communities. Because food is central to culture and wellness in the Hawaiian community, many of the KKNOW programs focus on promoting food sovereignty. KKNOW also houses the Waimānalo Pono Research Hui (WPRH), which is a community–academic partnership that promotes community-driven and culturally-grounded research (Keaulana et al. 2019). The word “pono” loosely translates in English to “righteous, goodness, uprightness, and moral qualities.” Pono is a sense of being and a way to work, act, respect, and treat people and the land to create balance and harmony. Pono aligns well with community-based participatory research principles (Israel et al. 2017; Israel et al. 2005) and Indigenous methodologies (Smith 2012), which guides the operation of this group. The members are composed of around 30 Waimānalo residents, university researchers, and students of all ages, who meet every month to continually shape the research and programming being implemented in Waimānalo. The members of the Waimānalo Pono Research Hui receive regular updates about the MALAMA study and provide feedback on its progress and future direction.

### 2.2. Participants

A total of ten Native Hawaiians families (each with 2–4 members) from the community of Waimānalo were recruited into the MALAMA Study. Criteria for inclusion were being a part of a Native Hawaiian family, aged 18 and older, Waimānalo resident, living in a home with sufficient outdoor space to keep the aquaponics system, and committed to maintaining their system for at least three months. To recruit participants, a flier was developed and distributed to various Native Hawaiian organizations and groups to invite them to a recruitment meeting. The recruitment meeting was held in the evening in Waimānalo and dinner was served. The participant requirements and eligibility criteria were explained during this meeting. To be mindful of Native Hawaiian collectivistic values, children under the age of 18 were invited to participate with adult family members in the workshops but were not considered as research participants. Participants signed a written informed consent form at the beginning of the study, which was also verbally explained to them. Costs of building and installing the aquaponics systems were entirely covered by the grant.

### 2.3. Measures

We used multiple measures to assess the feasibility and acceptability of the MALAMA intervention. We documented retention and attrition by tracking attendance for each gathering. Although the sample size was expected to be small and the intervention length of time was short, we also examined potential health benefits of the MALAMA intervention by administering a self-reported behavioral health questionnaire that included questions asking participants how many servings of fruit, vegetables, and fish they ate per day (Thompson et al. 2002). Although this was a pilot feasibility and we did not expect clinical changes due to the short timeframe of the intervention, we collected body composition measures to assess the feasibility and acceptability of collecting clinical data, including participants’ body mass index (BMI), blood pressure, and waist-to-hip ratio (WHR). Measurements were collected by trained clinicians employed by the University of Hawai’i John A. Burns School of Medicine (NHANES III 2022; Pickering et al. 2005). The health questionnaire and the clinical data were collected at three time points: before the intervention, immediately after the intervention, and three months after the intervention. At the end of the intervention, we also conducted a focus group with our participants to assess their satisfaction with and impacts of the intervention. Examples of questions were, “What did you like about the MALAMA Program?” and “Do you feel that the MALAMA Program integrated Native Hawaiian values and practices?”.

### 2.4. Procedures

After participants consented to being a part of the study, a day was scheduled where the participants arrived at a common location to complete the health questionnaire and clinical data. “Clinical stations” were set up around the area where clinicians collected the clinical measures of each participant. Participants moved around to the different stations and also completed the online survey using available iPads, laptops, or their personal phones. The questionnaire was completed online and took approximately 20–30 min to complete. The questionnaire was delivered using a web-based survey and data collection tool, REDCap. The clinical data collection took 30 min to complete. To promote cultural relevance and a family-friendly setting, meals were provided as well as a hands-on cultural activity.

Over the next three months, participants attended seven hands-on workshops together as a hui, where they learned how to build and maintain home backyard aquaponics. These workshops also embraced the Indigenous knowledge that “food is medicine” by integrating culturally-grounded teachings, such as ‘ai pono (healthy eating) and lā’au lapa’au (traditional spiritual medicine). Workshops also focused on gardening (planting, growing, and harvesting plants), growing and caring for fish, pest control, etc. Most workshops were approximately 2 to 3 h long. Towards the end of the three months, the program culminated with a Build Weekend, where the 10 families collectively built and installed a backyard aquaponics system in each other’s backyards.

The same health questionnaire and clinical data were collected after the last MALAMA workshop and three months later. At the last workshop, participants completed an hour-long audio recorded focus group, which was co-facilitated by an outside evaluator and a staff member. Participants received cultural gifts worth about $15 for participating in the focus group and each time they completed the questionnaire and the clinical data. This study was first reviewed and approved by the members of the Waimānalo Pono Research Hui to ensure community approval and then submitted to the University of Hawai’i Institutional Research Board, which approved it.

### 2.5. Analysis

Descriptive statistics were used to summarize the participants’ characteristics and clinical outcomes. Mean and standard deviation were calculated for continuous variables and frequencies and percentages were calculated for the categorical variables. Differences in clinical outcomes between pre- and post-intervention, post- and follow-up, and pre- and follow up were evaluated by paired *t* tests with Holm adjustment for multiple testings. The distribution of the clinical outcomes was assessed visually using scatter plot and histogram for checking outliers and the normality assumption of paired *t* test. The Kolmogorov–Smirnov test and Shapiro–Wilk test were also conducted to evaluate the normality assumption. All the analyses were performed using SAS 9.4 with significance level at 0.05.

The focus group data was audio-recorded and transcribed verbatim. Three coders independently completed an initial review of the transcript and identified emerging codes. Initial codes were entered into NVivo to code the transcript in detail and come to consensus on the codebook. Using flexible coding (Deterding and Waters 2021), emerging codes were identified and grouped into themes through consensus coding (Patton 2002; Strauss and Corbin 1990).

## 3. Results

### 3.1. Attendance

A total of 21 participants from 10 Native Hawaiian families enrolled in the MALAMA study (see Table 1). Research participants’ ages ranged from 18 to 68 years old, with a mean of 45 years. Most participants lived in multi-generational households with an average of six individuals per household, ranging from two to nine individuals per household. There were more male participants (57%) compared to female participants (43%).

**Figure 1.**
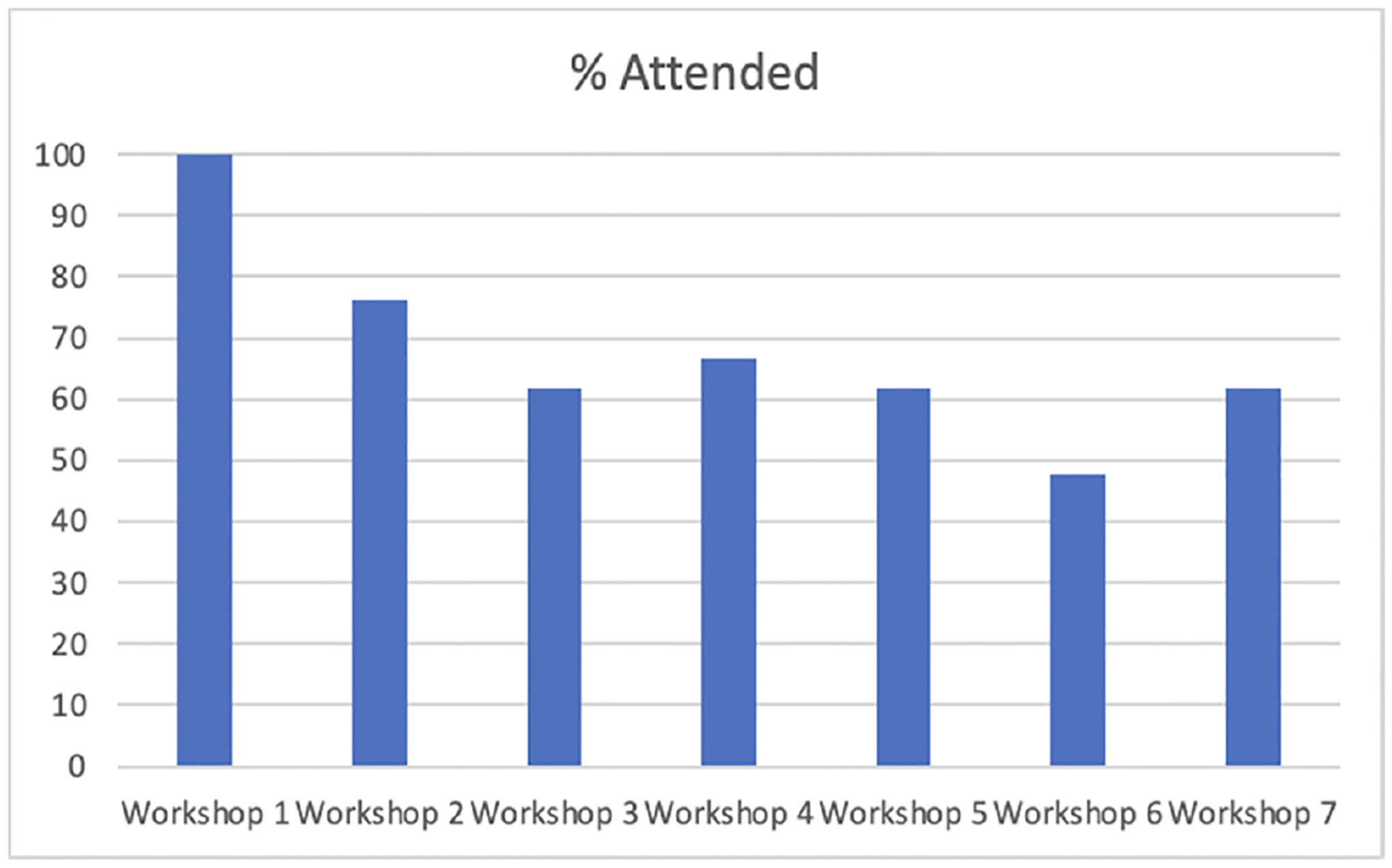
Proportion of participants who attended MALAMA by workshop.

A total of seven workshops were held during the MALAMA program. On average, 68% of the 21 study participants attended each session with a range of 10–21 participants per workshop (see Figure 1). At least one person from each family attended every session as required by our program. There was no attrition with no participant dropping out of the study over the three months. Approximately six keiki (children) attended each session with their families. The keiki were children, grandchildren, nieces, or nephews of the study participants.

### 3.2. Behavioral and Clinical Outcomes

Differences in the primary outcomes, eating habits, blood pressure, WHR (waist-to-hip ratio), and BMI (body mass index) were analyzed between three timepoints: before intervention (time 1), after intervention (time 2), and a 3-month follow up (time 3) (see Table 2). Fruit consumption among all participants significantly increased from time 1 (2.1 servings) to time 2 (2.9 servings). As expected, since this was a pilot feasibility study, no statistically significant differences in BMI and WHR were observed between any time points among participants, although there were favorable trends in blood pressure and fish and vegetable consumption.

### 3.3. Qualitative Themes

Five themes emerged from the focus group: (1) Coming together as a community; (2) Building family cohesion; (3) Valuing food sustainability and self-sufficiency; (4) Perpetuating a Hawaiian lifestyle; (5) Strengthening MALAMA for the future.

#### 3.3.1. Theme 1: Coming Together as a Community

Participants repeatedly shared how they appreciated being able to build new relationships within their hui. Because the MALAMA program is structured so that 10 families participate as a hui and move through all the hands-on workshops together, many felt that it was a valuable opportunity to get to know one another. Even though Waimānalo is considered a small community, people noted that everyone is busy working and going through their everyday lives. Being a part of the MALAMA Program allowed people to pause and connect with their neighbors through shared experiences and values. As a participant shared, “It’s kind of like we started off as strangers within our own community. Which is kind of embarrassing but we’re all so busy working and everything. Everybody work work work, you’ve got to be a millionaire to live in Hawai’i. So we form our lives working and making money. So this program gave us the opportunity to slow down, learn some Hawaiian stuff, sustainable growing that we can pass on to our families”. Another participant also spoke about the importance of relationships in building their confidence in learning new skills. She stated, “You think you can’t do it until you come to a group and we’re all doing it together.” The relationships built also enhanced their confidence in turning to each other in the future if they needed help. “So it’s kind of like we’re all family now. So I can come to you and ask you for help and you guys can count on me. But before this we just drove by each other.”

#### 3.3.2. Theme 2: Building Family Cohesion

Because MALAMA is a family-based program, the participants also spoke about how participating in MALAMA gave them an opportunity to spend time together as a family and strengthen their relationships. As a participant shared, “What I liked about it is that me and my family participated. And I have my husband here. And I was working and with this MALAMA program, he was here all the time and so that was great.” Another participant talked about how she appreciated the multigenerational learning and connection that was fostered within the family. “What I really liked about that was that I brought my two grandsons and they participated and they would have done more if they didn’t have to work. So I really enjoyed that, bringing our families, meeting all your families, being able to chit chat and the babies and all of that because I’m a family person I like being around my family.”

#### 3.3.3. Theme 3: Connecting to Food and Health

Participants discussed how participating in MALAMA made them feel more connected to food and the types of food they consume and how that impacts their health. As one participant shared, “I think for me it made me more aware of where my food comes from and what I put in my body…. To think that I got more education so that I can live sustainably in my own home. To think so, if anything were to happen, we can survive at least for a certain amount of time with our own fish, our own fruits, our own vegetables. So it made me just more aware of things that can empower me… This aquaponics program just made me more aware of the connection to what I put in my body.” Another participant agreed and stated, “It made me more ma’a (accustomed to, familiar, knowledgeable of) to what I need to bring into my life, made me more connected to the food.”

#### 3.3.4. Theme 4: Perpetuating a Hawaiian Lifestyle

Participants expressed that the MALAMA program and growing food with their aquaponic systems resonated with their Hawaiian culture and lifestyle. Hawaiian culture promotes sustainability and self-sufficiency, which many agreed were components that were emphasized in the MALAMA program. As one participant pointed out the similarities between aquaponics and the ahupua’a system, “It’s like sustainable living pieces. Right now we’re doing aquaponics, which in my own opinion is smaller down-sized version of true ahupua’a living.” Another participant pointed out that MALAMA goes beyond just being a program; it perpetuates the Hawaiian lifestyle. “I think it’s actually a lifestyle. It’s not just a program, but it should be into everything you do. The way you clean your hale, your house, yourself, your body, your mind, I think it’s not just getting together. That’s a big part of it, but a lot of it is what you’re doing at home because it should really be a lifestyle because after a while you want to start doing more things with aquaponics like what else can you plant? What else can you sustain yourself with?”

#### 3.3.5. Theme 5: Strengthening MALAMA for the Future

When asked how the MALAMA Program could be improved, the majority of the participants noted that the program length (3 months) was too short. As one participant pointed out, “Yeah I didn’t really have any negative things except what everyone else said, it was too short and compressed. The program was a little short and it could be stretched out a little more.” Another participant agreed and asked, “Can we get just a little more time? Because now that our plants are growing now, we’re experiencing bugs.” The participants also agreed that the clinical indicators collected were not invasive and acceptable and appreciated the opportunity to check on their health status.

## 4. Discussion

The findings of this pilot study suggest that MALAMA is a feasible and acceptable public health intervention for Native Hawaiian families living in Waimānalo. There was a high level of engagement and no participant attrition. In addition, a good proportion of our sample were male despite the pervasive challenge of recruiting males into studies (McDonald et al. 2020). Because of the short duration and small sample size, there were no statistically significant changes in clinical indicators. However, we did see an improving trend in blood pressure among participants. Clinical changes were not expected due to the short 3-month duration of the MALAMA program and the fact that the program focuses on improving long-term health benefits through holistic approaches. Previous studies that aimed to improve anthropometric measures, such as body mass index (BMI), body fat percentage, blood pressure, and WHR, document improvement after a typical 6-month intervention that focuses directly on nutrition and exercise (Johnson et al. 2016). However, these interventions lack long term changes. In fact, participants have been documented to return to pre-intervention measures after a two-year follow up (Rojo-Tirado et al. 2021). A robust literature review conducted in 2016 suggested that behavior change and family-based approaches are more effective in improving BMI and body fat percentage, especially among high-risk communities (Johnson et al. 2016). Consequently, we found statistically significant improvements in health behavior after three months. More specifically, participants increased consumption of healthier foods, such as fruit, fish, and vegetables. Although these findings are preliminary and require more investigation, they may point to the promising approach of MALAMA as a holistic and culturally-grounded way of improving health behavior, and later, health outcomes.

Although our quantitative results found limited evidence of health changes, our qualitative findings illuminated the health benefits of MALAMA with the majority of the participants identifying multiple positive impacts. One of the prevalent themes was the value of relationships. Participants noted that MALAMA gave them an opportunity to strengthen their relationships with other community members, their own family members, to the food that they grow and eat, and to the Hawaiian cultural lifestyle. Relationships, or pilina, hold great importance in Native Hawaiian health (Coleman et al. 2023; Odom et al. 2019). Building relationships and social connectedness between neighbors can be an important factor for sustainability and encourage participants to continue to meet on their own, even after the program is complete. From a Native Hawaiian perspective, health and wellness is holistic and interconnected with family, land, and community (McGregor et al. 2003). This is also aligned with Indigenous food sovereignty movement that goes beyond the concept of food security (Cidro et al. 2015). Food sovereignty is defined as “the right of peoples to healthy and culturally appropriate food produced through ecologically sound and sustainable methods, and their right to define their own food and agriculture systems” (U.S. Food Sovereignty Alliance 2022). The MALAMA program incorporates these concepts by reviving traditional methods of food production and environmental stewardship. Since the pilot study, an interactive MALAMA curriculum, which includes a series of demonstration videos that can be accessed online, PowerPoint slides, and laminated reference guides, has been developed to teach participants about aquaponics technology, planting and harvesting plants, fish health, and water quality. The curriculum also integrates Native Hawaiian stories, metaphors, language, and protocol and addresses the impact of historical trauma on dismantling the traditional food system and emphasizing traditional foods and traditional ways of knowing.

The participants provided other valuable insights to inform the future of MALAMA. For example, the short duration of the program was a prevalent theme. This recommendation is also practical given that the fish and plants take a few months to grow and be ready for consumption. It could also be one of the reasons why statistically significant changes may not have been found. This recommendation has been taken into consideration and therefore, the MALAMA curriculum is now longer in duration and the research design includes a longer follow-up. We also plan to gather and integrate feedback from our Lima Kōkua to help determine the feasibility of MALAMA.

## 5. Limitations

Given that this was a pilot study, our sample size was small, which impacts power and effect size. We also had a relatively heterogeneous sample with a wide age range. While age is strongly associated with high blood pressure, our sample size was not big enough to adjust for age. With such a small sample size, it is crucial to ensure that the methods used for quantitative analyses are suitable and yield reliable results. However, our primary aim was to assess the feasibility of the MALAMA study, as there has been no similar study conducted in Hawai’i. We plan to use these descriptive and quantitative results as a foundation for future, larger studies. Thus, we have been focusing our efforts on implementing MALAMA to more Native Hawaiian communities throughout Hawai’i and expanding our sample size, which will allow us to conduct more robust analysis to ensure the robustness and reliability of our findings in subsequent research.

## 6. Conclusions

Community-driven and culturally-grounded solutions, such as MALAMA, may be a promising approach to addressing pervasive health disparities and promoting health equity in minority and Indigenous communities.

## Figures and Tables

**Table 1.** Participant demographics, *N* = 21.

Demographic Factors	*n* (%)
Age, Mean(SD)	45 (15.3)
Household size, Mean(SD)	6 (2.7)
Sex, *n* (%)	
Male	12 (57%)
Female	9 (43%)
Education, *n* (%)	
High school/GED	5 (24%)
Some college/vocational	8 (38%)
College degree	7 (33%)
Graduate degree	1 (5%)

**Table 2.** Clinical measures and average servings of food consumed per day.

	Mean ± SD (n)			*p*-Value		
	Pre-Survey	Post-Survey	Follow-Up	Pre vs. Post	Post vs. Follow-Up	Pre-Follow-Up
Blood pressure systolic	122.81 ± 17.69 (16)	123.56 ± 16.52 (16)	120.67 ± 18.95 (9)	0.509	0.352	0.352
Blood pressure diastolic	78.94 ± 11.15 (16)	78.62 ± 12.85 (16)	74.33 ± 13.55 (9)	0.828	0.195	0.195
Waist-to-hip ratio	0.89 ± 0.09 (16)	0.90 ± 0.08 (16)	0.92 ± 0.09 (8)	0.774	>0.999	>0.999
BMI	31.16 ± 5.16 (16)	31.31 ± 5.12 (16)	31.83 ± 6.77 (8)	>0.999	>0.999	>0.999
Vegetables	2.40 ± 1.50 (15)	3.07 ± 2.25 (15)	3.33 ± 1.41 (9)	0.359	>0.999	>0.999
Fruit	2.07 ± 1.10 (15)	2.87 ± 1.60 (15)	3.11 ± 1.36 (9)	0.049	0.665	0.499
Fish	1.53 ± 1.36 (15)	2.00 ± 1.36 (15)	2.78 ± 1.92 (9)	0.755	0.149	0.755

BMI = Body Mass Index; The *p*-values calculated two-sided paired *t*-tests with Holm adjustment for multiple comparisons, *p* < 0.05 considered significant.

## Data Availability

Data is owned by Ke Kula Nui O Waimānalo. The original contributions presented in the study are included in the article, further inquiries can be directed to the corresponding author.
